# *N*-(3,5-Dichloro­phen­yl)benzamide

**DOI:** 10.1107/S1600536808017017

**Published:** 2008-06-07

**Authors:** B. Thimme Gowda, Sabine Foro, B. P. Sowmya, Hartmut Fuess

**Affiliations:** a Department of Chemistry, Mangalore University, Mangalagangotri 574 199, Mangalore, India; b Institute of Materials Science, Darmstadt University of Technology, Petersenstrasse 23, D-64287 Darmstadt, Germany

## Abstract

The conformation of the H—N—C=O unit in the title compound, C_13_H_9_Cl_2_NO, is *trans*, similar to the conformation observed in *N*-(3-chloro­phen­yl)benzamide, *N*-(2,3-dichloro­phen­yl)benzamide, *N*-(2,4-dichloro­phen­yl)benzamide, *N*-(2,6-dichloro­phen­yl)benzamide and *N*-(3,4-dichloro­phen­yl)benz­amide. The amide group makes dihedral angles of 14.3 (8) and 44.4 (4)° with the benzoyl and aniline rings, respectively, while the benzoyl and aniline rings form a dihedral angle of 58.3 (1)°. The mol­ecules are linked by N—H⋯O hydrogen bonds into infinite chains running along the *c* axis.

## Related literature

For related literature, see: Gowda *et al.* (2003 ▶, 2007 ▶, 2008*a* ▶,*b* ▶).
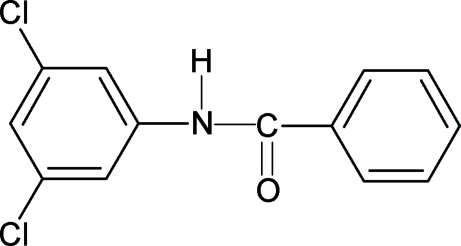


## Experimental

### 

#### Crystal data

C_13_H_9_Cl_2_NO
*M* *_r_* = 266.11
Monoclinic, 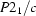

*a* = 13.520 (1) Å
*b* = 9.9929 (8) Å
*c* = 9.4447 (7) Å
β = 106.357 (9)°
*V* = 1224.37 (16) Å^3^
*Z* = 4
Mo *K*α radiation
μ = 0.51 mm^−1^
*T* = 299 (2) K
0.48 × 0.36 × 0.26 mm

#### Data collection

Oxford Diffraction Xcalibur diffractometer with Sapphire CCD detector
Absorption correction: multi-scan (*CrysAlis RED*; Oxford Diffraction, 2007 ▶) *T*_min_ = 0.792, *T*_max_ = 0.879
7774 measured reflections
2493 independent reflections
1837 reflections with *I* > 2σ(*I*)
*R*_int_ = 0.013

#### Refinement

*R*[*F* ^2^ > 2σ(*F* ^2^)] = 0.037
*wR*(*F* ^2^) = 0.133
*S* = 1.12
2493 reflections
181 parameters
Only H-atom coordinates refined
Δρ_max_ = 0.44 e Å^−3^
Δρ_min_ = −0.29 e Å^−3^

### 

Data collection: *CrysAlis CCD* (Oxford Diffraction, 2007 ▶); cell refinement: *CrysAlis RED* (Oxford Diffraction, 2007 ▶); data reduction: *CrysAlis RED*; program(s) used to solve structure: *SHELXS97* (Sheldrick, 2008 ▶); program(s) used to refine structure: *SHELXL97* (Sheldrick, 2008 ▶); molecular graphics: *PLATON* (Spek, 2003 ▶); software used to prepare material for publication: *SHELXL97*.

## Supplementary Material

Crystal structure: contains datablocks I, global. DOI: 10.1107/S1600536808017017/bt2720sup1.cif

Structure factors: contains datablocks I. DOI: 10.1107/S1600536808017017/bt2720Isup2.hkl

Additional supplementary materials:  crystallographic information; 3D view; checkCIF report

## Figures and Tables

**Table 1:** Hydrogen-bond geometry (Å, °)

*D*—H⋯*A*	*D*—H	H⋯*A*	*D*⋯*A*	*D*—H⋯*A*
N1—H1*N*⋯O1^i^	0.83 (3)	2.18 (3)	2.964 (2)	157 (2)

Symmetry code: (i) 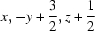.

**Fig. 1.**
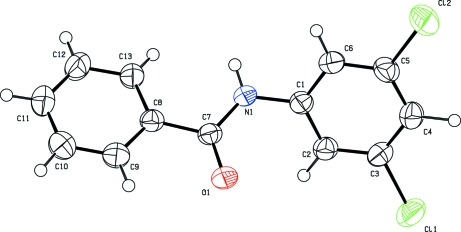
Molecular structure of the title compound, showing the atom labeling scheme. The displacement ellipsoids are drawn at the 50% probability level. H atoms are represented as small spheres of arbitrary radii.

**Fig. 2.**
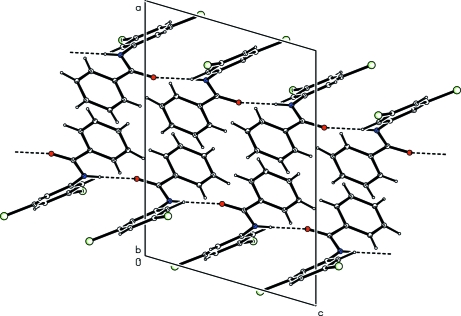
Molecular packing of the title compound with hydrogen bonding shown as dashed lines.

## supplementary crystallographic information

### Comment

In the present work, the structure of *N*-(3,5-dichlorophenyl)-benzamide (N35DCPBA) has been determined to explore the effect of substituents on the solid state geometries of benzanilides (Gowda *et al.*, 2003, 2007, 2008*a*, 2008*b*). The conformation  of the H-N-C=O unit in is trans (Fig. 1), similar to that observed in *N*-(3-chlorophenyl)-benzamide (Gowda *et al.*, 2008*a*), *N*-(2,3-dichlorophenyl)-benzamide and *N*-(3,4-dichlorophenyl)- benzamide (Gowda *et al.*, 2007), *N*-(2,4-dichlorophenyl)-benzamide and *N*-(2,6-dichlorophenyl)-benzamide(Gowda *et al.*, 2008*b*). The amide group –NHCO– makes the dihedral angles of 14.3 (8)° and 44.4 (4)° with the benzoyl and aniline rings, respectively, while the benzoyl and aniline rings form the dihedral angle of 58.3 (1)°). Part of the crystal structure of the title compound with infinite molecular chains running along the *c* axis is shown in Fig. 2. The chains are generated by N—H···O hydrogen bonds (Table 1)

### Experimental

The title compound was prepared according to the literature method (Gowda *et al.*, 2003). The purity of the compound was checked by determining its melting point. It was characterized by recording its infrared and NMR spectra. Single crystals of the title compound were obtained from an ethanolic solution and used for X-ray diffraction studies at room temperature.

### Refinement

The H atoms were located in a difference map, and their positional parameters were refined freely with U(H) set to 1.2U_eq_ of the parent atom.

### Crystal data

**Table:**

C_13_H_9_Cl_2_NO	*F*_000_ = 544
*M**_r_* = 266.11	*D*_x_ = 1.444 Mg m^−^^3^
Monoclinic, *P*2_1_/*c*	Mo *K*α radiation λ = 0.71073 Å
Hall symbol: -P 2ybc	Cell parameters from 3450 reflections
*a* = 13.520 (1) Å	θ = 2.4–28.1º
*b* = 9.9929 (8) Å	µ = 0.51 mm^−^^1^
*c* = 9.4447 (7) Å	*T* = 299 (2) K
β = 106.357 (9)º	Prism, colourless
*V* = 1224.37 (16) Å^3^	0.48 × 0.36 × 0.26 mm
*Z* = 4	

### Data collection

**Table:**

Oxford Diffraction Xcalibur diffractometer with Sapphire CCD detector	2493 independent reflections
Radiation source: fine-focus sealed tube	1837 reflections with *I* > 2σ(*I*)
Monochromator: graphite	*R*_int_ = 0.013
*T* = 299(2) K	θ_max_ = 26.4º
Rotation method data acquisition using ω and φ scans	θ_min_ = 2.6º
Absorption correction: multi-scan(CrysAlis RED; Oxford Diffraction, 2007)	*h* = −12→16
*T*_min_ = 0.792, *T*_max_ = 0.879	*k* = −12→12
7774 measured reflections	*l* = −11→11

### Refinement

**Table:**

Refinement on *F*^2^	Secondary atom site location: difference Fourier map
Least-squares matrix: full	Hydrogen site location: difference Fourier map
*R*[*F*^2^ > 2σ(*F*^2^)] = 0.037	Only H-atom coordinates refined
*wR*(*F*^2^) = 0.133	*w* = 1/[σ^2^(*F*_o_^2^) + (0.0671*P*)^2^ + 0.5267*P*] where *P* = (*F*_o_^2^ + 2*F*_c_^2^)/3
*S* = 1.12	(Δ/σ)_max_ = 0.010
2493 reflections	Δρ_max_ = 0.44 e Å^−^^3^
181 parameters	Δρ_min_ = −0.29 e Å^−^^3^
Primary atom site location: structure-invariant direct methods	Extinction correction: none

### Special details

**Table:**

Experimental. (CrysAlis RED; Oxford Diffraction, 2007) Empirical absorption correction using spherical harmonics, implemented in SCALE3 ABSPACK scaling algorithm.
Geometry. All e.s.d.'s (except the e.s.d. in the dihedral angle between two l.s. planes) are estimated using the full covariance matrix. The cell e.s.d.'s are taken into account individually in the estimation of e.s.d.'s in distances, angles and torsion angles; correlations between e.s.d.'s in cell parameters are only used when they are defined by crystal symmetry. An approximate (isotropic) treatment of cell e.s.d.'s is used for estimating e.s.d.'s involving l.s. planes.
Refinement. Refinement of *F*^2^ against ALL reflections. The weighted *R*-factor *wR* and goodness of fit *S* are based on *F*^2^, conventional *R*-factors *R* are based on *F*, with *F* set to zero for negative *F*^2^. The threshold expression of *F*^2^ > σ(*F*^2^) is used only for calculating *R*-factors(gt) *etc*. and is not relevant to the choice of reflections for refinement. *R*-factors based on *F*^2^ are statistically about twice as large as those based on *F*, and *R*- factors based on ALL data will be even larger.

### Fractional atomic coordinates and isotropic or equivalent isotropic displacement parameters (Å^2^)

**Table:**

	*x*	*y*	*z*	*U*_iso_*/*U*_eq_	
C1	0.18869 (16)	0.6454 (2)	0.5651 (2)	0.0370 (5)	
C2	0.11990 (16)	0.6605 (2)	0.4259 (2)	0.0389 (5)	
H2	0.1002 (18)	0.753 (2)	0.384 (3)	0.047*	
C3	0.07367 (17)	0.5473 (2)	0.3519 (2)	0.0412 (5)	
C4	0.09118 (18)	0.4206 (2)	0.4131 (3)	0.0452 (5)	
H4	0.0558 (19)	0.332 (3)	0.354 (3)	0.054*	
C5	0.15740 (19)	0.4104 (2)	0.5533 (3)	0.0446 (5)	
C6	0.20746 (18)	0.5197 (2)	0.6309 (2)	0.0429 (5)	
H6	0.2544 (19)	0.506 (3)	0.726 (3)	0.051*	
C7	0.29164 (16)	0.8484 (2)	0.5806 (2)	0.0357 (4)	
C8	0.34903 (16)	0.9572 (2)	0.6776 (2)	0.0358 (5)	
C9	0.4140 (2)	1.0369 (3)	0.6232 (3)	0.0525 (6)	
H9	0.419 (2)	1.022 (3)	0.530 (4)	0.063*	
C10	0.4693 (2)	1.1390 (3)	0.7066 (3)	0.0628 (7)	
H10	0.509 (2)	1.189 (3)	0.663 (3)	0.075*	
C11	0.4590 (2)	1.1653 (3)	0.8447 (3)	0.0601 (7)	
H11	0.497 (2)	1.235 (3)	0.898 (3)	0.072*	
C12	0.3940 (2)	1.0892 (3)	0.8994 (3)	0.0573 (7)	
H12	0.379 (2)	1.110 (3)	0.989 (3)	0.069*	
C13	0.33965 (19)	0.9850 (2)	0.8168 (2)	0.0450 (5)	
H13	0.296 (2)	0.942 (3)	0.852 (3)	0.054*	
N1	0.24216 (15)	0.75725 (18)	0.64286 (19)	0.0395 (4)	
H1N	0.2559 (19)	0.753 (2)	0.734 (3)	0.047*	
O1	0.29162 (14)	0.84106 (17)	0.45101 (16)	0.0518 (4)	
Cl1	−0.00774 (6)	0.56249 (7)	0.17406 (7)	0.0640 (2)	
Cl2	0.18142 (6)	0.25339 (6)	0.63392 (8)	0.0716 (3)	

### Atomic displacement parameters (Å^2^)

**Table:**

	*U*^11^	*U*^22^	*U*^33^	*U*^12^	*U*^13^	*U*^23^
C1	0.0448 (11)	0.0354 (10)	0.0335 (10)	−0.0010 (9)	0.0157 (9)	−0.0022 (8)
C2	0.0456 (12)	0.0359 (11)	0.0347 (10)	0.0036 (9)	0.0103 (9)	0.0004 (9)
C3	0.0435 (12)	0.0443 (12)	0.0335 (10)	0.0036 (9)	0.0071 (9)	−0.0037 (9)
C4	0.0516 (13)	0.0381 (12)	0.0453 (12)	−0.0011 (10)	0.0129 (10)	−0.0066 (10)
C5	0.0552 (13)	0.0331 (11)	0.0468 (12)	0.0031 (10)	0.0163 (10)	0.0038 (9)
C6	0.0512 (13)	0.0432 (12)	0.0325 (10)	0.0001 (10)	0.0090 (9)	0.0027 (9)
C7	0.0421 (11)	0.0355 (10)	0.0295 (9)	0.0026 (9)	0.0103 (8)	0.0006 (8)
C8	0.0390 (11)	0.0354 (11)	0.0329 (10)	0.0029 (8)	0.0098 (8)	0.0016 (8)
C9	0.0641 (16)	0.0570 (15)	0.0413 (12)	−0.0134 (12)	0.0226 (11)	−0.0022 (11)
C10	0.0679 (17)	0.0681 (18)	0.0550 (15)	−0.0263 (14)	0.0213 (13)	0.0010 (13)
C11	0.0656 (16)	0.0569 (16)	0.0548 (15)	−0.0226 (13)	0.0120 (12)	−0.0103 (12)
C12	0.0697 (17)	0.0625 (16)	0.0418 (13)	−0.0181 (13)	0.0195 (12)	−0.0142 (12)
C13	0.0530 (13)	0.0470 (13)	0.0383 (11)	−0.0120 (11)	0.0181 (10)	−0.0051 (10)
N1	0.0544 (11)	0.0387 (10)	0.0257 (8)	−0.0073 (8)	0.0120 (8)	−0.0025 (7)
O1	0.0769 (11)	0.0517 (10)	0.0306 (8)	−0.0128 (8)	0.0215 (7)	−0.0043 (7)
Cl1	0.0752 (5)	0.0601 (4)	0.0419 (3)	0.0005 (3)	−0.0076 (3)	−0.0028 (3)
Cl2	0.0945 (6)	0.0372 (3)	0.0729 (5)	0.0021 (3)	0.0066 (4)	0.0131 (3)

### Geometric parameters (Å, °)

**Table:**

C1—C2	1.389 (3)	C7—C8	1.491 (3)
C1—C6	1.392 (3)	C8—C13	1.385 (3)
C1—N1	1.419 (3)	C8—C9	1.387 (3)
C2—C3	1.383 (3)	C9—C10	1.375 (4)
C2—H2	1.01 (2)	C9—H9	0.91 (3)
C3—C4	1.384 (3)	C10—C11	1.375 (4)
C3—Cl1	1.736 (2)	C10—H10	0.91 (3)
C4—C5	1.377 (3)	C11—C12	1.369 (4)
C4—H4	1.09 (3)	C11—H11	0.92 (3)
C5—C6	1.382 (3)	C12—C13	1.381 (3)
C5—Cl2	1.734 (2)	C12—H12	0.95 (3)
C6—H6	0.95 (3)	C13—H13	0.87 (3)
C7—O1	1.226 (2)	N1—H1N	0.83 (3)
C7—N1	1.358 (3)		
			
C2—C1—C6	120.66 (19)	C13—C8—C9	118.3 (2)
C2—C1—N1	120.79 (19)	C13—C8—C7	123.98 (19)
C6—C1—N1	118.54 (19)	C9—C8—C7	117.74 (18)
C3—C2—C1	118.4 (2)	C10—C9—C8	120.9 (2)
C3—C2—H2	121.4 (14)	C10—C9—H9	119.7 (19)
C1—C2—H2	120.0 (14)	C8—C9—H9	119.5 (19)
C2—C3—C4	122.5 (2)	C11—C10—C9	120.1 (2)
C2—C3—Cl1	119.37 (17)	C11—C10—H10	123 (2)
C4—C3—Cl1	118.13 (17)	C9—C10—H10	116 (2)
C5—C4—C3	117.3 (2)	C12—C11—C10	120.0 (2)
C5—C4—H4	120.5 (14)	C12—C11—H11	121.7 (19)
C3—C4—H4	122.3 (14)	C10—C11—H11	118 (2)
C4—C5—C6	122.7 (2)	C11—C12—C13	120.1 (2)
C4—C5—Cl2	118.68 (18)	C11—C12—H12	122.4 (19)
C6—C5—Cl2	118.63 (18)	C13—C12—H12	117.3 (19)
C5—C6—C1	118.4 (2)	C12—C13—C8	120.7 (2)
C5—C6—H6	118.9 (17)	C12—C13—H13	118.0 (18)
C1—C6—H6	122.7 (17)	C8—C13—H13	121.0 (18)
O1—C7—N1	122.05 (19)	C7—N1—C1	123.09 (16)
O1—C7—C8	120.68 (18)	C7—N1—H1N	119.4 (17)
N1—C7—C8	117.25 (17)	C1—N1—H1N	115.4 (17)
			
C6—C1—C2—C3	−2.5 (3)	O1—C7—C8—C9	−9.3 (3)
N1—C1—C2—C3	176.84 (19)	N1—C7—C8—C9	169.2 (2)
C1—C2—C3—C4	1.9 (3)	C13—C8—C9—C10	1.3 (4)
C1—C2—C3—Cl1	−176.86 (16)	C7—C8—C9—C10	180.0 (2)
C2—C3—C4—C5	0.1 (3)	C8—C9—C10—C11	−1.4 (4)
Cl1—C3—C4—C5	178.90 (17)	C9—C10—C11—C12	0.2 (5)
C3—C4—C5—C6	−1.6 (4)	C10—C11—C12—C13	0.8 (5)
C3—C4—C5—Cl2	−179.98 (17)	C11—C12—C13—C8	−0.8 (4)
C4—C5—C6—C1	1.0 (3)	C9—C8—C13—C12	−0.2 (4)
Cl2—C5—C6—C1	179.39 (17)	C7—C8—C13—C12	−178.8 (2)
C2—C1—C6—C5	1.1 (3)	O1—C7—N1—C1	1.4 (3)
N1—C1—C6—C5	−178.3 (2)	C8—C7—N1—C1	−177.06 (19)
O1—C7—C8—C13	169.3 (2)	C2—C1—N1—C7	−47.9 (3)
N1—C7—C8—C13	−12.2 (3)	C6—C1—N1—C7	131.4 (2)

### Hydrogen-bond geometry (Å, °)

**Table:**

*D*—H···*A*	*D*—H	H···*A*	*D*···*A*	*D*—H···*A*
N1—H1N···O1^i^	0.83 (3)	2.18 (3)	2.964 (2)	157 (2)

Symmetry codes: (i) *x*, −*y*+3/2, *z*+1/2.
